# Mutual Information between Order Book Layers

**DOI:** 10.3390/e24030343

**Published:** 2022-02-27

**Authors:** Daniel Libman, Gil Ariel, Mary Schaps, Simi Haber

**Affiliations:** Department of Mathematics, Bar-Ilan University, Ramat Gan 5290002, Israel; arielg@math.biu.ac.il (G.A.); mschaps@macs.biu.ac.il (M.S.); simi.haber@biu.ac.il (S.H.)

**Keywords:** limit order book, entropy, entropy estimation, mutual information, mutual information estimation, recursive copula, deep layers of order book, price and volume

## Abstract

The order book is a list of all current buy or sell orders for a given financial security. The rise of electronic stock exchanges introduced a debate about the relevance of the information it encapsulates of the activity of traders. Here, we approach this topic from a theoretical perspective, estimating the amount of mutual information between order book layers, i.e., different buy/sell layers, which are aggregated by buy/sell orders. We show that (i) layers are not independent (in the sense that the mutual information is statistically larger than zero), (ii) the mutual information between layers is small (compared to the joint entropy), and (iii) the mutual information between layers increases when comparing the uppermost layers to the deepest layers analyzed (i.e., further away from the market price). Our findings, and our method for estimating mutual information, are relevant to developing trading strategies that attempt to utilize the information content of the limit order book.

## 1. Introduction

Stock exchanges provide an organized platform for traders to exchange securities, which is known as the limit order book. Traders use orders as a tool to indicate their willingness to buy or sell an instrument in the market. Generally, orders are one of two types: bid (indicating willingness to buy) and ask (indicating willingness to sell). Each order can further be classified as one of two main categories: limit and market. A limit order is an agreement to buy (or sell) a given number of shares of a particular stock at a given price (or better). This type of order is usually not fulfilled immediately and is instead listed in the limit order book while waiting for a match. In contrast, market orders are orders to buy or sell at the best currently available price in the limit order book. Since these orders have no price restrictions, market orders are typically fulfilled instantaneously.

Every order is recorded in the limit order book, and when a match between a buyer and a seller occurs, the exchange executes an exchange of securities—a trade—and the corresponding orders are removed from the book. At any point in time, there may be outstanding orders to buy or sell a certain amount of a security at different price points. These price points can be thought of as the layers of the order book. Overall, the time evolution of the limit order book encapsulates an enormous amount of information, which includes all of the financial actions of all traders, including both fulfilled and unfulfilled orders.

**Figure 1:**
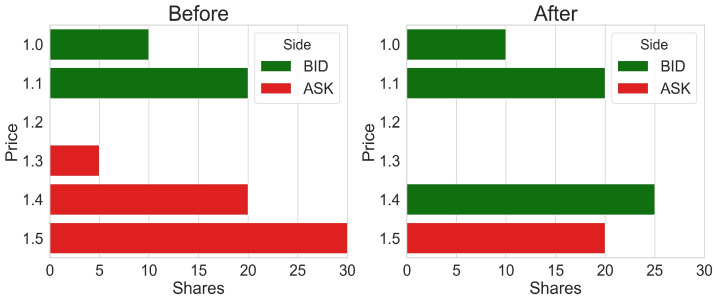
An example illustrating order book dynamics. An order to buy 50 shares at limit price 1.4 is submitted. This order results in 5 shares being exchanged at price 1.3, 20 shares exchanged at price 1.4, and the remaining 25 shares in the order are now waiting in the order book at price 1.4 on the bid side. As a result, the bid layer shifted from 1.1 vol. 20 to 1.4 vol. 25.

The highest price at which buyers are willing to buy a security and the lowest price at which sellers are willing to sell a security collectively comprise the “bid–ask” layers, which are the uppermost layers and represent the current market price of a security. Additional layers are price points that are further away from the bid–ask. Each layer consists of a price and volume. The bid–ask layers change continuously throughout the day based on supply and demand, resulting in shifts in the security’s market price. For instance, a flow of buy orders can exhaust the volume available in the uppermost ask layer. This would uncover the next available layer on the ask side, making this layer the new ask market layer and thereby raising the stock price. Thus, the limit order book contains hidden data that may become visible throughout the trading day (see Figure 1 for an example).

The ability of a market to sustain a large order while avoiding a significant change in price is called the market depth, and it is considered a proxy for the liquidity of the market. Deep markets enjoy a large number and volume of orders waiting for execution in the different layers. Conceptually, market depth summarizes the state of the different layers of the limit order book and thus may act as one mechanism for the information leak we have discovered between the layers.

Some of the orders populating the deeper layers have a specific nature. Stop losses are limit orders placed far from the bid–ask layer and in the opposite direction of the trader’s belief of price change. Larger orders may be put relatively deep to soothe their influence on the price. Slower traders sometimes use a limit order on the medium-distanced layers to mitigate their inability to control for short-term variance in price. In all of these cases, traders express their expectations for the price in the deeper layers. Our results coincide with this view of shared information between layers, increasing with depth.

In addition to and rising from their primary role as a trading platform, stock exchanges serve multiple additional functions. One of these involves price determination, e.g., the market pricing of a certain security at a given time. For example, Alan and Schwartz [1] studied the impact of exchange factors, such as trading volatility, on stock price discovery. Sirignano and Cont [2] showed that the price movements of a given security can be predicted from the price history and order flow of other securities, suggesting that the exchanges have a role in price formation.

The layers of the limit order book that are further away from the market price play a role in price determination. Information recorded in the deeper layers of the order book, beyond the market bid–ask layers, has been typically hidden from most traders and reserved for specific groups or types of traders, which are generally more sophisticated or experienced. However, as stock exchanges around the world have shifted to an electronic format, sharing the data from the deeper layers became more practical. This introduced a debate about the value of the information contained in the deeper layers. The debate is still the subject of active research. For example, Harris [3] suggests that specialists leverage information from the deeper layers when placing trades. Bloomfield [4] finds that the number of limit orders increases when traders have access to information about the deeper layers. In addition, Madhavan [5] discovered that traders at the Toronto Stock Exchange placed fewer orders after the top four layers became visible to all traders.

These studies have focused mostly on the stock market. However, studies of the FX market paint a different picture. For example, Kozhan and Salmon [6] showed that in the Dollar Sterling market, although variables such as depth, spread, and order flow can explain returns, incorporating these data into trading strategies does not yield profits that are statistically significant from those obtained in trading without this information on hand. Meanwhile, Gençay and Gradojevic [7] show that up to 25% of the variation in the FX market can be explained by private trader information, implying that information in the order book indeed has limited utility in this market. Gradojevic et al. [8] show that although the limit order book can be useful in the FX market, its efficacy can quickly disappear for arbitrage traders in a highly volatile market. The authors contend that in such scenarios, arbitrage traders are likely to be more successful by using liquidity measures. Kozhan and Tham [9] also research arbitrage traders and found that factors such as the number of market participants as well as speed have a substantial impact on execution risk, including resulting profits and/or loss from trades. Thus, different aspects of the market may come into play for different trading scenarios.

The debate surrounding the information content of the limit order book is associated with a practical one, namely, how many layers of the limit order book should be exposed to traders by the exchanges. “Information” here means anything that affects the distribution of the measured quantity. Different agents in the market may have an interest in different segments of the information content of the limit order book. Since the bid–ask layers play a major role in determining future prices, this information is relevant for all traders. Anyone who wishes to execute large orders should take the deeper layers into account; hence, the information in these layers is of particular importance for a large volume of players.

Research on the deeper layers of the limit order book generally suggests that the deeper layers include some information. For instance, Libman et al. [10] showed that compared to the uppermost bid–ask layers, using information from the deeper layers improves accuracy in predicting the log quoted depth, which is a measure of liquidity. Cao [11] concluded that data from the deeper layers promotes price discovery, while Baruch [12] claims that the NYSE’s open limit order book benefits traders.

In this paper, we address a more basic question—how much new information is contained in the deep layers, if at all? We decided to look at this question in the context of smaller exchanges. For this paper, we worked with the Tel Aviv Stock Exchange (TASE). Generally, the limit order book in small exchanges repopulates slowly (e.g., the order book has low resilience), which underscores the importance of studying the layer depth.

Rather than measuring the efficacy of the deep layers in forecasting of particular trading measures, we examine the mutual information (MI) between the layers. Entropy and MI have been previously applied in financial data, as described in the a review by Zhou et al. [13]. Specifically, Cai et al. [14] and Almog and Shmueli [15] use entropy to study the effect of auto-correlations in stock and FOREX time-series. Avellaneda [16] and Avellaneda et al. [17] used minimum relative entropy to fine-tune pricing models. Sakalauskas and Kriksciuniene [18] combined Shannon’s entropy with Hurst exponents to forecast changes in stock price trends, while Kim et al. [19] combined effective transfer entropy with machine learning models to forecast changes in the direction of stock prices. In Dionisio et al. [20] and Darbellay and Wuertz [21], MI is applied to stock market indexes. Two papers specifically studied the MI between securities traded on the NYSE (Fiedor [22]) and Shanghai Stock Exchange (Guo et al. [23]). Both works found that the MI method yields different results compared to correlation coefficients. These findings suggest the existence of nonlinear relationships in financial markets.

To the best of our knowledge, the current study is one of the early papers that addresses the information content in the limit order book. Our results indicate that the amount of MI increases with layer depth, and therefore, deeper layers have a higher degree of similarity to each other. This implies that the amount of new information offered by each layer decreases as depth increases; e.g., as we descend deeper into the order book, each layer reveals less new information than the one preceding it. Our findings suggest that not all of the deeper layers might be equally of interest to traders.

## 2. Methodology

Our contribution involves calculating the entropy between the order book layers. To accomplish this, we used the trading data of thirty-five securities traded on the Tel Aviv Stock Exchange (TASE) in 2017. To make our analysis practical, we were compelled to select stocks that had sufficient trading activity and thus resembled stocks in larger exchanges. For this reason, we focused our analysis on stocks featured in the TA-35, which represents the 35 most actively traded stocks with the highest market capitalization on the Tel Aviv Stock Exchange. For clarity, we show the full analysis for five of these stocks, aiming to select a variety of industries, ranging from technology to banking, real estate, and consumer products. Then, we list the summarized results for all thirty-five stocks in the index.

The trading activity dataset, which was provided directly by TASE, was comprised of one text file for all order submissions (including cancelled orders) and another text file for executed transactions. The files were separated by security and date. Table 1 shows several summary statistics for each of the five securities.

Each of the stocks had a minimum price increment, or interval, defined by the TASE. This meant that orders could only be placed at specific price points. For instance, if the increment was 0.10 Israeli Shekels (ILS) and the market price was 7.50, the next price layer would be at 7.40 from the bid side or 7.60 from the ask side. Thus, since the prices were subject to constraints by the exchange, the price variable on both the bid and ask side would always originate from a finite set. For our calculations, we used the number of increments from the uppermost layer, e.g., best bid–ask, rather than the nominal price point itself. This provided additional uniformity in the price. These characteristics of the price allowed us to regard the price as a discrete variable.

**Figure 2:**
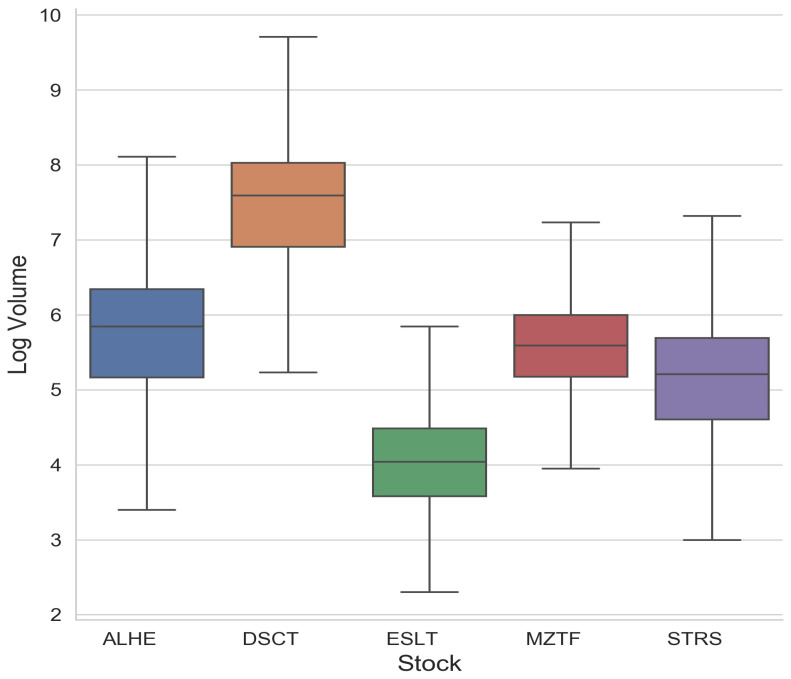
Order log(volume) statistics.

**Figure 3:**
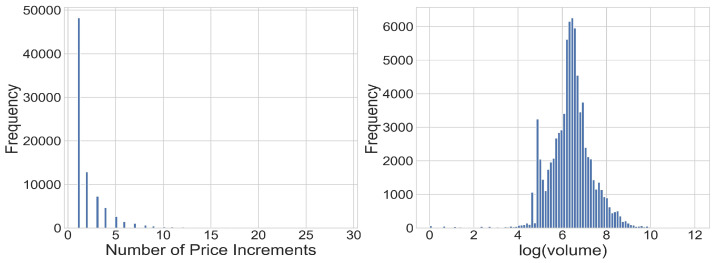
An example of the distribution of price increments and log(volume) for one order book layer of ALHE in 2017. The number of price increments are a count of the minimum interval in price set by TASE from the best bid and best ask. For instance, if the best bid is 7 and the increment is 0.10, a price of 7.30 would appear as 3. This illustrates that price differences are discrete, having specific values with some rare ones, while the log(volume) is approximately continuous in nature.

The volume data behaved far differently than the price. Since there were few restrictions on the volume of orders, the volume could change freely between the layers, and indeed, the volume data included a wide variety of different values. For this reason, we regarded the volume as a continuous variable. In order to be certain that the volume resembled a continuous distribution, we added some random noise uniformly distributed between zero and one to the log volume dataset. To validate that the noise did not contribute to the results, we also ran the same analysis using a different noise that was normally distributed and had a standard deviation of one. This ensured that no two values were exactly the same, while the data integrity remained intact. For our calculations, we used the natural log of the volume. This helped normalize the dataset and is supported by the fact that orders placed in the financial markets generally follow a power law. See, for example, Zovko and Farmer [24]. The statistics of the log volume of the orders for each security can be seen in Figure 2. Figure 3 shows a histogram of price differences between the layers and the log volume.

The information from the TASE orders was used to recreate the dynamics of the limit order book, classifying each incoming order to the bid or ask side, while keeping a record of the previous orders and executing a transaction whenever a match occurred. As a benchmark, we compared the trading output from our simulation to the actual transaction records and verified the two were identical. Next, we proceeded to capture the order book layers’ status after every transaction. Since accounting for factors such as intraday seasonality and day-of-the-week effects would have meant splitting the data into smaller batches which would have rendered the high-dimensional entropy estimation inaccurate in our case, we opted to run the analysis on data pooled from all times of day and week.

These snapshots were created for the bid and ask sides separately, yielding a snapshot of the order book sorted by price points, or layers. In order to validate the consistency of the observed patterns, results were compared to a diluted time series in which every other snapshot (or two out of three) were discarded. No qualitative differences were observed.

A major challenge in measuring the entropy of the order book layers was the fact that each layer of the book is described by side (e.g., bid or ask), volume, and price. Since our data are comprised of discrete and continuous variables (e.g., price and volume, respectively), we split the dataset based on the different values of the discrete random variable and used the conditional entropy to sum the entropies.

We can describe a snapshot of the order book by setting *G*={bid,ask} and α∈G. Using this formulation, we can define $v_{k}^{\alpha }\left(t\right)$ and $p_{k}^{\alpha }\left(t\right)$ as the log(volume) and price difference, respectively, for layer *k* after transaction *t*. Thus, the log(volume) for the bid side in the first layer after the first transaction can be represented as $v_{1}^{\mathrm{bid}}\left(1\right)$. Similarly, the price difference for the ask side on the second layer after the third transaction of the day can be represented as $p_{2}^{\mathrm{ask}}\left(3\right)$.

The full snapshot of the first five layers of the book after transaction *n* can be represented using a vector in $R^{20}$ as follows:

(1) $$S\left(t\right)=(v_{k}^{\alpha }\left(t\right),p_{k}^{\alpha }\left(t\right)|k=1\ldots 5,\alpha \in G)$$

The mutual information between two order book layers *i* and *j* can be separated into three contributions using the entropy of the layers as follows:

(2) I(i,j)=H(i)+H(j)−H(i,j)

Each of these components, e.g., H(i), H(j), and H(i,j), can be represented as a conditional entropy that is conditioned on the price, which is discrete in nature. Thus, calculating H(i) can be completed by defining $X:(p_{i}^{\mathrm{bid}}\left(n\right),p_{i}^{\mathrm{ask}}\left(n\right))\mapsto R$ and $Y\in (v_{i}^{\mathrm{bid}}\left(n\right),v_{i}^{\mathrm{ask}}\left(n\right))$ as follows (note that different *Y* values correspond to different *X* values):

(3) $$H\left(i\right)=H\left(X\right)+\sum_{k}P\left(X=k\right)H\left(\vec{Y}|X=k\right)$$

Calculating the difference in price entropy H(X) was accomplished using the method proposed by Valiant and Valiant [25], who provide a method for estimating discrete entropy when some parts of the distribution are rare and therefore undersampled, which is a phenomenon we encountered in the price data. Indeed, this method was a good fit for our case, since our analysis showed that differences in prices tend to have some rare outliers that are difficult to measure.

Evaluating $\sum_{k}P\left(X=k\right)H\left(Y|X=k\right)$ is also non-trivial. While the probability of *X* to be a certain value can be easily estimated by counting the occurrences divided by the length of the dataset, it is not practical to measure H(Y|X=k) for all values of *X*. Doing so would have generated some filters with very limited data counts that are insufficient for reliable estimation of the entropy for the continuous part. Instead, we split the the data into three equal-size groups based on the *X* values. This was achieved by estimating the cumulative distribution function of *X* and splitting three intervals $l_{1},l_{2},l_{3}$ such that for ∀i $P\left(X\in l_{i}\right)=\frac{1}{3}$. The results for each of the different groups is detailed in Appendix A, Table A1.

Calculating $H(Y|X\in l_{i})$, which is the entropy for a multi-dimensional continuous random variable comprised of the log volume data after filtering to $l_{i}$, can be done using the recursive method described by Ariel and Louzoun in [26].

The general idea underlying the algorithm in [26] is to apply the Sklar’s theorem, which states that any continuous distribution $R^{D}$ can be decomposed as

(4) $$p\left(x\right)=p_{1}\left(x_{1}\right)p_{2}\left(x_{2}\right)\ldots p_{D}\left(x_{d}\right)c\left(F_{1}\left(x_{1}\right),F_{2}\left(x_{2}\right),\ldots ,F_{D}\left(x_{d}\right)\right),$$

where $p_{i}\left(x_{i}\right)$ are the marginal distributions of $x_{i}$, $F_{i}\left(x_{i}\right)$ are the commutative distribution functions, and $c(u_{1}\ldots u_{D})$ is the joint density, which is called the copula. Substituting H=−∫p(x)lnp(x)dx into the definition of the continuous entropy yields,

(5) $$H=\sum_{k=1}^{D}H_{k}+H_{c},$$

where $H_{k}$ is the one-dimensional entropy of the marginal distributions, which is straightforward to calculate using one of the numerical estimation methods described in Berilant et al. [27].

Estimating $H_{c}$, the copula entropy, is more complex. For this, Ariel and Louzoun [26] proposed a recursive method that involves splitting the length of the dimensions into statistically independent blocks. Then, for each of these blocks, the method involves performing a change of variables similar to an integral transformation, which uses the actual data to transform each of the points over each dimension to its empirical CDF value. This value can be defined as $\hat{F}\left(t\right)=\frac{1}{n}\sum_{i}1_{x_{i}<t}$. Then, the transformed dataset is split into two groups along the median of one of the dimensions. Next, the recursive part reruns the entropy estimation of each one of the two subsets. This continues recursively and essentially converts the original problem into a series of summations on one-dimensional entropies. The Python code we have used is provided in [28] as open source under GPL.

Ariel and Louzon [26] showed that unlike other algorithms, where performance might differ among distributions, their method is fairly accurate for a wide family of distributions. Other advantages of the method included a relatively low complexity and ability to converge fast at the order O(DNlog(N)) where *N* is the sample size.

For each one of our experiments, we rejected the hypothesis that the mutual information equals zero. This was accomplished by computing $I^{\prime }\left(i,j\right)$, which is the mutual information after creating a random permutation of the data in layer *j*. We repeated this experiment 1000 times for each of the layers under examination and calculated the ratio of times for which we obtained a higher value, $P_{value}=\#(I^{\prime }\left(i,j\right)>I\left(i,j\right)/1000$. This numerical *p*-value indicates the probability of achieving at least the number we obtained in a random setting. The mutual information obtained in the shuffled data was far lower and the pattern seen with the real data was not visible. The results are given in Appendix A, Table A1.

Checking for the significance of the increase in the mutual information between the upper layers of the book and the deeper ones was done using a Student’s *t*-test for the mean of paired samples, where we checked the hypothesis that there was an increase in the mean of the MI when comparing the two uppermost layers to the two deepest layers, e.g., the mean of the MI of the two deepest layers is higher.

To verify the stability of the results and ensure that the way we sampled the book after each transaction did not impact the results, we repeated the analysis using three different configurations for the sampling of the order book layers. The first took a snapshot after every transaction, the second took a snapshot after every two transactions, and the third took a snapshot after every three transactions. All three configurations yielded the same phenomena. We took this as evidence against the hypothesis that sampling the snapshots after each transaction affected the results.

We had sufficient data to calculate the entropy for the first five layers of the book; e.g., we had five layers of data in every snapshot. However, we realized that particularly in a small stock exchange such as the TASE, we sometimes may not have data for deeper layers, which would make entropy estimation difficult (if not impractical). For this reason, we decided not to extend the analysis to deeper layers. However, since the work with the five stocks indicated that the significance of new layers was declining substantially by layer 5, we expect that deeper layers will behave similarly.

A more detailed analysis of the full entropy calculation, including by groups of price differences, is presented in Appendix A, Table A1.

## 3. Results

**Figure 4:**
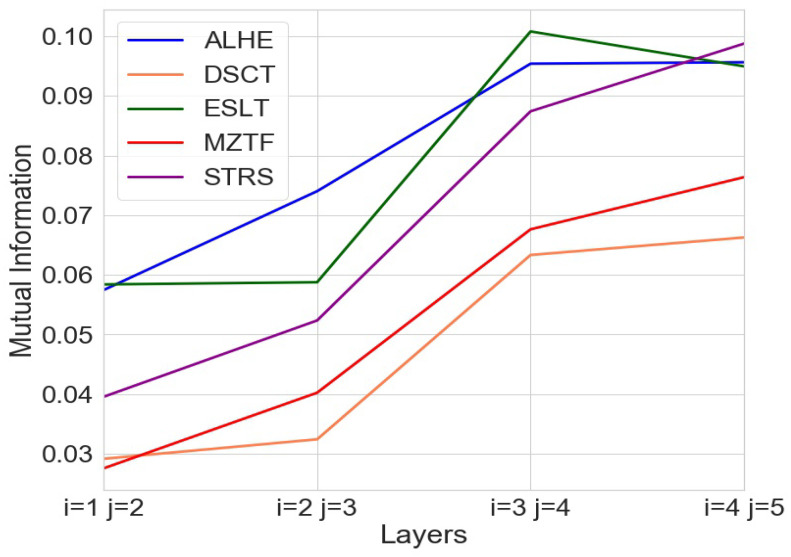
MI of different layers when capturing a snapshot after one transaction.

**Figure 5:**
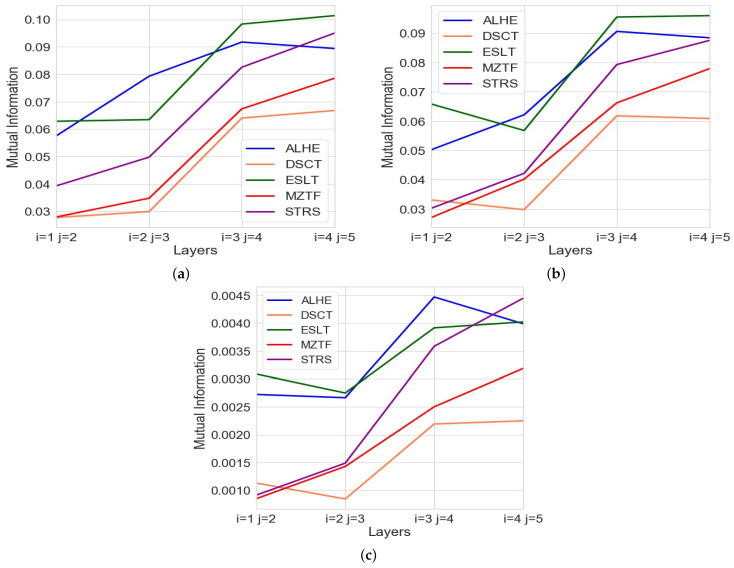
MI of different layers with varying the noise as well as the number of transactions between snapshots. (**a**) MI of layer i and j after two transactions. (**b**) MI of layer i and j after three transactions. (**c**) MI of different layers when capturing a snapshot after one transaction using a normal distributed noise instead of a uniform noise.

Figure 4 shows the mutual information (MI) between different layers for each of the five stocks when calculated after every transaction. As mentioned above, we also ran the same analysis with a lag of two and three transactions; see Figure 5a,b, respectively. Figure 5c demonstrates that these results remain similar when repeating the analysis with different noise, as described above in the Methodology section.

Our results show that as we dive deeper into the limit order book, the mutual information between the layers increases. This can be seen in all of the figures. The stability of the findings across every transaction as well as multiple transactions further validates our findings.

In addition, to further support these observations, we calculated Student’s *t*-test for the mean of paired samples where we checked the hypothesis that there was an increase in the mean of the MI between the deepest layers and the uppermost layers. The results of the analysis are shown in Table 2. We see a high statistical significance for the hypothesis that the MI is higher for the deepest layers vs. the uppermost layers. This significance exists across all of the three configurations of the order book snapshots. After completing the shuffling described previously, we counted the number of times that the MI calculation on the shuffled data was higher than the one calculated with real data. In the shuffled data, the MI was far smaller, yielding a very low *p*-value. Table 3 contains the results of our analysis on shuffled data, suggesting that our findings were statistically significant.

We also extended Student’s *t*-test for the mean of paired samples to all of the TA-35 stocks. The full mutual information results are shown in Appendix A, Table A2, and the statistical analysis can be seen in Table 4. The results show that the phenomena observed with the initial smaller set is also significant across all of the TA-35 stocks.

The results describe strong evidence that as we dive deeper into the order book, the layers become more similar, and the new information gained from each additional layer decreases.

## 4. Discussion

The results in this contribution show that the amount of mutual information slightly increases as the layer depth increases, suggesting that each successive layer of the limit order book is more similar to the one that preceded it. The results withstand statistical significance tests. Future research can apply our methods to more stocks in additional stock markets, particularly prominent ones such as the New York Stock Exchange (NYSE). Such research would also benefit from accounting for factors such as intraday seasonality and day-of-the-week effects, which were impractical for our dataset as mentioned above. Nevertheless, we believe that these findings are relevant for any researcher attempting to evaluate the relevance of the deeper layers of the limit order book. We also believe that these findings should be considered by stock exchanges when determining whether to expose the content of the deeper layers of the limit order book to all traders and if so, how many layers to reveal. Since the content of the order book layers changes dynamically based on human behavior, further research can also compare the mutual information in open and closed markets, e.g., exchanges where the deeper order book layers are visible to all traders, some traders, or not at all. Additional factors such as exchange size and location might prove worthwhile to analyze, as well. 

## Figures and Tables

**Table 1:** Statistical summary of raw orders and transactions data prior to grouping into layers. Values are presented as median (±IQR/2). Volume in count of instruments. Data include all trading days in 2017.

Security Ticker	Order Volume (Instruments)	Transaction Volume (Instruments)
ALHE	346 (±196)	186 (±143)
DSCT	1985 (±1031)	1156 (±769)
ESLT	57 (±26)	25 (±18)
MZTF	268 (±113)	172 (±107)
STRS	183 (±98)	100 (±61)

**Table 2:** A *t*-test for the mean of paired samples analysis checking for the increase in MI between deepest layers compared to the uppermost layers. The lag indicates the three different configurations used to select the order book snapshots: after one transaction, two transactions, and three transactions.

lag	t_stat	*p*-Value
1	9.943	0.000287
2	9.895	0.000293
3	7.117	0.001030

**Table 3:** The MI of layers *i* and *j*. The *p*-value of each MI calculation was estimated by shuffling the value of layer *j* 1000 times and counting the number of times that the MI calculation was higher than the one calculated with actual data.

Stock	ALHE		DSCT		ESLT		MZTF		STRS	
Layers	MI	pv	MI	pv	MI	pv	MI	pv	MI	pv
*i* = 1 *j* = 2	0.057	<0.01	0.029	<0.01	0.058	<0.01	0.0276	<0.01	0.040	<0.01
*i* = 2 *j* = 3	0.074	<0.01	0.032	<0.01	0.059	<0.01	0.040	<0.01	0.052	<0.01
*i* = 3 *j* = 4	0.095	<0.01	0.063	<0.01	0.101	<0.01	0.068	<0.01	0.087	<0.01
*i* = 4 *j* = 5	0.096	<0.01	0.066	<0.01	0.095	<0.01	0.076	<0.01	0.099	<0.01

**Table 4:** A *t*-test for the mean of paired samples analysis checking for the increase in MI between the deepest layers and the uppermost layers using all of the TA-35 stocks.

N	t_stat	*p*-Value
35	6.166	<0.0001

## Data Availability

The dataset involves limit order book trading data from the Tel Aviv Stock Exchange (TASE). Requests to access these datasets should be directed to micky@tase.co.il.

## Appendix A

**Table A1:** The full entropy calculations for the initial five stocks.

Layers	Stock	H(Y)	H(X)	H(X,Y)	H-Groups	*p*-Value	AVG-MI-Permutation
X = (4) Y = 5	ALHE	3.188	3.107	6.199	(6.14, 6.20, 6.25)	<0.01	−0.016
X = (3) Y = 4	ALHE	3.107	3.033	6.044	(5.97, 6.07, 6.10)	<0.01	−0.014
X = (2) Y = 3	ALHE	3.033	2.599	5.558	(5.48, 5.61, 5.59)	<0.01	−0.027
X = (1) Y = 2	ALHE	2.599	2.633	5.175	(5.02, 5.33, 5.16)	<0.01	−0.034
X = (4) Y = 5	DSCT	3.038	2.906	5.877	(6.05, 5.79, 5.79)	<0.01	−0.011
X = (3) Y = 4	DSCT	2.906	2.768	5.610	(5.75, 5.48, 5.60)	<0.01	−0.005
X = (2) Y = 3	DSCT	2.768	2.522	5.258	(5.29, 5.17, 5.31)	<0.01	−0.017
X = (1) Y = 2	DSCT	2.522	2.572	5.065	(4.96, 5.15, 5.08)	<0.01	−0.017
X = (4) Y = 5	ESLT	2.966	2.857	5.729	(5.68, 5.71, 5.80)	<0.01	−0.009
X = (3) Y = 4	ESLT	2.857	2.837	5.593	(5.50, 5.58, 5.70)	<0.01	−0.011
X = (2) Y = 3	ESLT	2.837	2.558	5.336	(5.28, 5.39, 5.34)	<0.01	−0.020
X = (1) Y = 2	ESLT	2.558	2.629	5.129	(5.04, 5.26, 5.11)	<0.01	−0.006
X = (4) Y = 5	MZTF	2.968	2.832	5.724	(5.79, 5.67, 5.71)	<0.01	−0.012
X = (3) Y = 4	MZTF	2.832	2.693	5.458	(5.45, 5.42, 5.50)	<0.01	−0.011
X = (2) Y = 3	MZTF	2.693	2.431	5.084	(5.03, 5.04, 5.19)	<0.01	−0.021
X = (1) Y = 2	MZTF	2.431	2.515	4.918	(4.78, 5.04, 4.93)	<0.01	−0.024
X = (4) Y = 5	STRS	3.116	2.975	5.993	(6.20, 6.00, 5.78)	<0.01	−0.024
X = (3) Y = 4	STRS	2.975	2.795	5.683	(5.77, 5.65, 5.63)	<0.01	−0.017
X = (2) Y = 3	STRS	2.795	2.503	5.245	(5.20, 5.20, 5.34)	<0.01	−0.021
X = (1) Y = 2	STRS	2.503	2.512	4.975	(4.80, 5.11, 5.01)	<0.01	−0.020

**Table A2:** The mutual information (MI) measured between layers of the order book for all of the TA-35 stocks. The columns indicate the layers mentioned. For example, the top left cell shows the MI between layers 1 and 2 for ALHE stock.

MI (*i*,*j*) Symbol	1_2	2_3	3_4	4_5
ALHE	0.057	0.074	0.095	0.096
AMOT	0.099	0.109	0.187	0.205
ARPT	0.084	0.092	0.124	0.121
AZRG	0.099	0.116	0.118	0.130
BEZQ	0.046	0.053	0.079	0.084
BIG	0.091	0.111	0.138	0.162
CEL	0.062	0.082	0.136	0.137
DEDR	0.153	0.271	0.257	0.228
DLEKG	0.072	0.084	0.092	0.092
DSCT	0.029	0.032	0.063	0.066
ESLT	0.058	0.059	0.101	0.095
FIBI	0.025	0.032	0.033	0.019
FRUT	0.078	0.111	0.158	0.138
GZT	0.062	0.080	0.104	0.113
HARL	0.093	0.080	0.125	0.133
ICL	0.088	0.091	0.128	0.141
ILCO	0.078	0.085	0.121	0.129
LUMI	0.283	0.415	0.502	0.490
MLSR	0.072	0.083	0.114	0.120
MYL	0.120	0.172	0.184	0.201
MZTF	0.028	0.040	0.068	0.076
NICE	0.042	0.067	0.106	0.110
NVMI	0.094	0.099	0.111	0.117
OPK	0.072	0.092	0.109	0.106
ORA	0.094	0.126	0.119	0.118
ORL	0.132	0.168	0.150	0.180
POLI	0.172	0.283	0.411	0.392
PRGO	0.124	0.127	0.148	0.130
PTNR	0.082	0.108	0.132	0.139
PZOL	0.062	0.077	0.136	0.144
SAE	0.153	0.133	0.174	0.168
SODA	0.093	0.078	0.138	0.133
STRS	0.040	0.052	0.087	0.099
TEVA	0.223	0.306	0.217	0.237
TSEM	0.214	0.189	0.123	0.150
